# Central Sleep Apnea Detection by Means of Finger Photoplethysmography

**DOI:** 10.1109/JTEHM.2023.3236393

**Published:** 2023-01-12

**Authors:** Frederik Massie, Steven Vits, Ani Khachatryan, Bart Van Pee, Johan Verbraecken, Jeroen Bergmann

**Affiliations:** Natural Interaction LabDepartment of EngineeringUniversity of Oxford 6396 OX1 2JD Oxford U.K.; Research Group LEMPFaculty of Medicine and Health Sciences, University of Antwerp 26660 2000 Antwerp Belgium; Ectosense 3000 Leuven Belgium; Medicine and Multidisciplinary Sleep Disorders CentreDepartment of PulmonaryAntwerp University Hospital 26660 2650 Edegem Belgium

**Keywords:** Sleep disorders, home sleep apnea testing, central sleep apnea, photoplethysmography, peripheral arterial tonometry

## Abstract

Obstructive Sleep Apnea (OSA) and Central Sleep Apnea (CSA) are two types of Sleep Apnea (SA) with different etiologies and treatment options. Home sleep apnea testing based on photoplethysmography-derived peripheral arterial tonometry (PAT HSAT) has become the most widely deployed outpatient SA diagnostic method. Being able to differentiate between CSA and OSA based solely on photoplethysmography-data would further increase PAT HSAT’s clinical utility. The present work proposes a method to detect CSA using finger photoplethysmography (PPG) data and evaluates the proposed method against simultaneous in-lab polysomnography (PSG). Methods: For 266 patients with a suspicion of SA, concurrent in-lab PSG and PPG data were acquired. The respiratory information embedded in the PPG data was extracted and used to train an ensemble of trees classifiers that predicts the central or obstructive nature of each respiratory event. The classifier performance was evaluated using patient-wise leave-one-out cross-validation where an expert analysis of the PSG served as ground truth. A second, independent analysis of the PSG was also evaluated against the ground truth to allow benchmarking of the PPG-based method. Results: The method achieved a sensitivity of 81%, a specificity of 99%, a positive predictive value of 90%, and a negative predictive value of 98% at the central apnea-hypopnea index cutoff of 10 events per hour of sleep. Conclusion and Significance: The present study aimed to evaluate a method to detect CSA in SA patients using only PPG data which could be used to flag CSA which in turn may aid in more optimal therapy decision making.

## I. Introduction

Sleep Apnea (SA) is a common sleep disorder affecting millions of people worldwide [1], [2]. An apnea event is defined as a cessation of airflow during sleep lasting 10 seconds or more, whereas a hypopnea event is characterized by an airflow reduction rather than a full cessation [3], [4]. A patient’s SA severity can be expressed by their apnea-hypopnea index (AHI), which is simply the number of apnea and hypopnea events per hour of sleep [5], [6], [7].

SA can be obstructive (OSA) or central (CSA) in nature. OSA is the more common form of SA, and some of its symptoms and consequences are fatigue, daytime sleepiness, cardiac arrhythmia, and systemic hypertension [3]. During OSA, breathing effort continues but the upper airway is mechanically obstructed, resulting in interruptions of airflow. CSA, on the other hand, is characterized by a lack of neural drive to breathe during sleep [8], [9]. Both types of respiratory events can coexist in one patient. A respiratory event can also be of mixed origin, characterized by a lack of respiratory drive followed by obstructive breathing. Typically, CSA is the primary diagnosis when at least 50% of respiratory events are scored as central in origin. CSA can be characterized by the cAHI (central AHI), which is calculated as the number of central respiratory events per hour of sleep.

Whereas the symptoms of CSA are often similar to those of OSA, the choice of therapy depends on the type of SA. Treatment of OSA includes lifestyle measures (for instance weight loss), mandibular advancement devices, surgical procedures and, most commonly, continuous positive airway pressure (CPAP) [10], [11]. Comparably, a wide variety of therapies for CSA exist of which some are distinctly different from therapies for OSA. These methods include drug intervention, oxygen therapy, nocturnal mechanical ventilation (via nasal mask or tracheostomy and tracheal tube), and diaphragm pacing [3], [12]. As such, for clinicians to make optimal therapy decisions, it is important to differentiate between CSA and OSA in SA patients.

**FIGURE 1.**
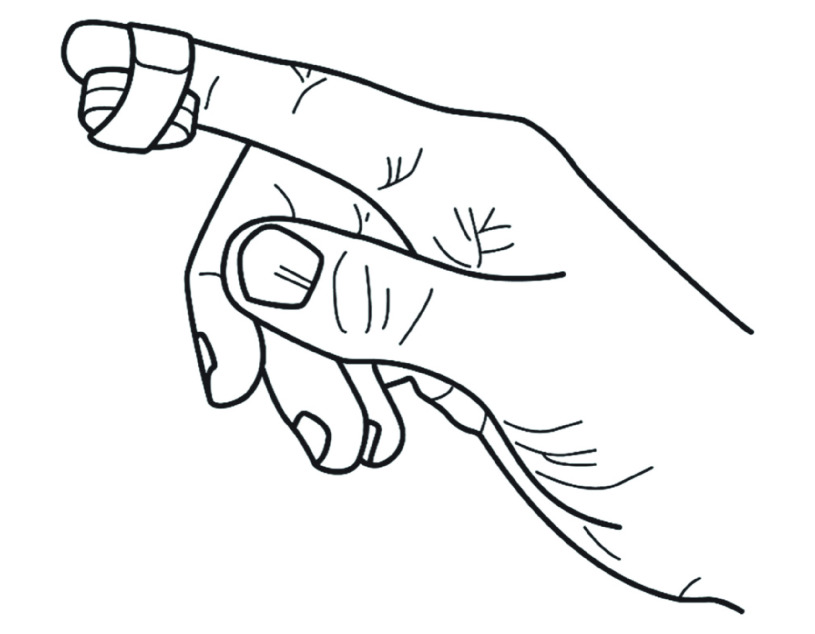
Illustration of the Study Device.

The gold standard for diagnosing SA is polysomnography (PSG) [13], [14]. PSG utilizes electroencephalography, electro-oculography, electromyography, electrocardiography, and pulse oximetry, as well as airflow and respiratory effort to assess underlying causes of sleep disturbances [15]. However, due to the inconvenience of performing an in-lab PSG and in part due to the global COVID pandemic, Home Sleep Apnea Testing (HSAT) based on peripheral arterial tonometry (PAT) have rapidly gained popularity and currently comprise the most widely deployed category of HSAT [16]. PAT HSAT allows for minimally invasive multi-night testing and is available in a fully disposable format [17], [18]. This study makes use of the FDA cleared NightOwl PAT HSAT (Study Device), first described by Massie et al. [19], [20]. It comprises a finger probe of the size of a fingertip and a cloud-based analysis software (Fig. 1).

Compared to PSG, PAT HSATs have a major drawback, namely a reduced channel design. Indeed, a PAT-based HSAT obtains most of its modalities from finger photoplethysmography (PPG), from which it derives the blood oxygen saturation (SpO_2_), pulse rate (PR), and PAT. Inherently, this means that PAT HSATs do not have access to information sources such as airflow reduction and cortical arousals and must instead rely on alternative sources of information to infer the presence of respiratory events [17].

**FIGURE 2.**
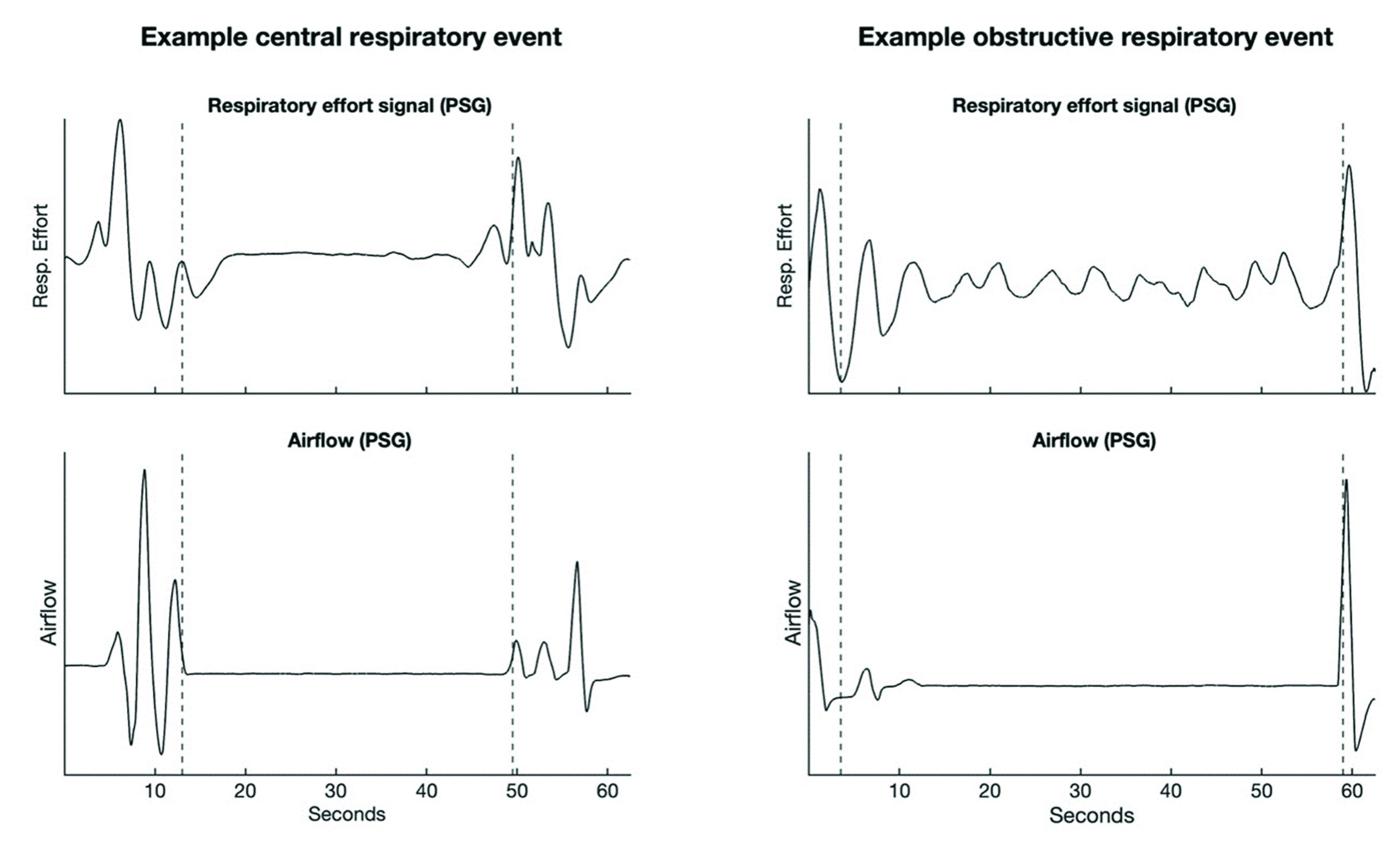
Comparison of a central and obstructive respiratory event. Both events are accompanied by cessation of airflow, but a central event is characterized by lack of respiratory effort which is reflected in a flat region of the respiratory effort belt signal. PSG = polysomnography.

One more challenge for PAT HSATs is the differentiation between the types of respiratory events. Both types of events are characterized by airflow reduction. However, central respiratory events are accompanied by a cessation of respiratory effort as traditionally inferred from the abdominal and thoracic respiratory effort belts of the PSG (which monitor respiratory-related fluctuations in the abdominal and thoracic circumference) [5]. Fig. 2 shows a comparison of the respiratory effort during a central and obstructive respiratory event.

The study device, utilizing only a finger PPG probe for respiratory event detection, does not have airflow and respiratory effort channels that can be utilized for respiratory event type labeling. The present study aims to evaluate a method to detect CSA in SA patients using only the PPG data acquired at the finger by the study device. A validated PPG-based CSA detection method could be used to flag CSA in patients which enables referral to an in-lab PSG for further confirmation.

The key idea behind the method is to extract and analyze respiratory-effort related information from the finger PPG data, as such deriving a signal that resembles PSG-based respiratory effort as closely as possible. This extracted breathing signal is then used to extract features to train and validate an ensemble of trees classifier that attempts to learn how to differentiate between the two types of respiratory events.

The present work is, to our knowledge, the first evaluation of an end-to-end approach for CSA detection based solely on finger PPG data with patient-wise performance evaluation.

## II. Related Works

Recently, Pillar et al. published a validation study of their novel CSA detection method using the WatchPAT PAT HSAT [21] with extra sensors placed on the chest. In addition to finger PPG, the system comprises an optional snoring and body position (SBP) sensor positioned under the sternal notch. The SBP sensor is used to derive upper chest wall respiratory movements that are diminished during central respiratory events. According to Pillar et al. [21], the PPG’s systolic upstroke shows more variability during obstructive respiratory events compared to central ones. The method uses both the upstroke variability together with the respiratory effort data acquired by the SBP sensor to identify central events.

Lazazzera et al. also published a method to detect and classify respiratory events into apnea and hypopnea and their origin (central or obstructive) using PPG and SpO_2_ signals [22]. Their method uses PPG features, SpO_2_ features, pulse rate time domain features, and pulse rate frequency domain features to classify respiratory events. However, they do not report on any patient-wise performance analysis, nor do they report on the cAHI estimation accuracy. As such, their method’s potential for clinical deployment remains unclear.

## III. Methods

### A. Pat HSAT and Its Principle of Operation

PAT HSAT makes use of a fingertip mounted PPG probe [19], [20]. PPG operates on simple optical technology to detect blood volume changes in the tissue’s microvascular bed [23], [24]. PPG measurements are used to derive the arterial blood oxygen saturation (SpO_2_), pulse rate (PR), and changes in peripheral arterial tone, which are then used to detect respiratory events [19], [20]. Peripheral arterial tone refers to the tone of the peripheral arterial smooth muscle tissue. When the muscle tone of peripheral arteries increases, the arteries’ diameter decreases, resulting in a reduction of perfusion and thus a decrease in pulsatile blood volume in the peripheral tissue. The latter is picked up as a drop in the PPG signal swing between systole and diastole. The PPG-derived signal, trending such pulsatile blood volume reductions, is referred to as the PAT signal. A PAT HSAT analyzes the concurrence of drops in SpO_2_, surges in pulse rate, and increases in peripheral arterial tone. As airflow is reduced, the oxygen supply to the lungs decreases from baseline, resulting in distinct SpO_2_ drops. Near the end of a respiratory event, the sympathetic nervous system activity spikes to arouse the patient, resulting in resumption of ventilation, a surge in pulse rate and a release of norepinephrine in the blood stream. Consequently, norepinephrine binds to alpha-adrenergic receptors innervating the arterial smooth muscle tissue in the finger, causing sudden vasoconstrictions, and corresponding increases in PAT, picked up by the PPG sensor [19], [20].

The same PPG sensor of the Study Device used to detect respiratory events is used in this study to extract respiratory effort-related information from which we attempt to discriminate between central and obstructive respiratory events.

### B. The Dataset

The dataset used in this study comprises 266 patients with suspicion of SA which were prospectively recruited across four different sleep clinic centers of which three were located in the USA (where all centers were part of the United Health Services Group in Miami, Florida) and one in Belgium (Ziekenhuis Oost Limburg, ZOL, Genk). The first patient was recruited on the $4^{\mathrm {th}}$ of July 2017 and recruitment was concluded on the $25^{\mathrm {th}}$ of January 2020. The USA branch of the study was approved by Aspire IRB, part of the WIRB-Copernicus Group. The Belgian branch of the study was approved by the Ethics Committee of ZOL.

All patients were scheduled for one overnight in-lab PSG. The patient cohort was not stratified for CSA prevalence to retain the normal cohort prevalence of CSA. Non-adult and/or mentally challenged patients were excluded. As the cohort matches the intended target population of the Study Device, this leads to the most representative prevalence-dependent performance endpoints such as Negative Predictive Values (NPV) and Positive Predictive Values (PPV).

Qualified lab technicians at each participating study center were responsible for setting up the equipment and capturing PSG data. During setup of PSG, the PAT HSAT was attached to the middle finger of the hand to which the pulse oximeter of PSG was applied.

All PSG data were double-scored by two independent centers which were blinded from one-another’s analysis. The first scoring was performed by the team of sleep technicians of the center where the patient was admitted (Local Analysis). Another independent scoring was performed by scorers of Cerebra Medical (CM, Canada). The studies were first analyzed by their Michele Sleep Scoring System (MSSS) and were subsequently complemented with complete manual rescoring by an expert technologist. Malhotra et. Al [25] confirmed in a multi-centric trial that the MSSS, complemented with manual editing by an expert scorer, is more robust than the results of a single scorer. Because of this conclusion, CM’s analysis served as the expert benchmark (Expert Analysis) to which the Local Analysis and our PPG-based method were compared. All PSG data were scored according to the latest AASM scoring rules using the recommended 1A (3%) rule for hypopnea scoring [26].

### C. Working Principles of the CSA Detection Method

#### 1) Respiratory Event Extraction from Raw PPG

The PAT HSAT uses PPG and PPG derived signals such as PR and SpO_2_ to detect respiratory events. This method has been previously validated by Massie et al. and Van Pee et al. [19], [20]. At this point, no distinction has been made between obstructive and central events. The labeling of respiratory events as central or obstructive for CSA detection model training is performed using PSG annotations (see section C. 4).

#### 2) Respiratory Signal Extraction from Raw PPG

**FIGURE 3.**
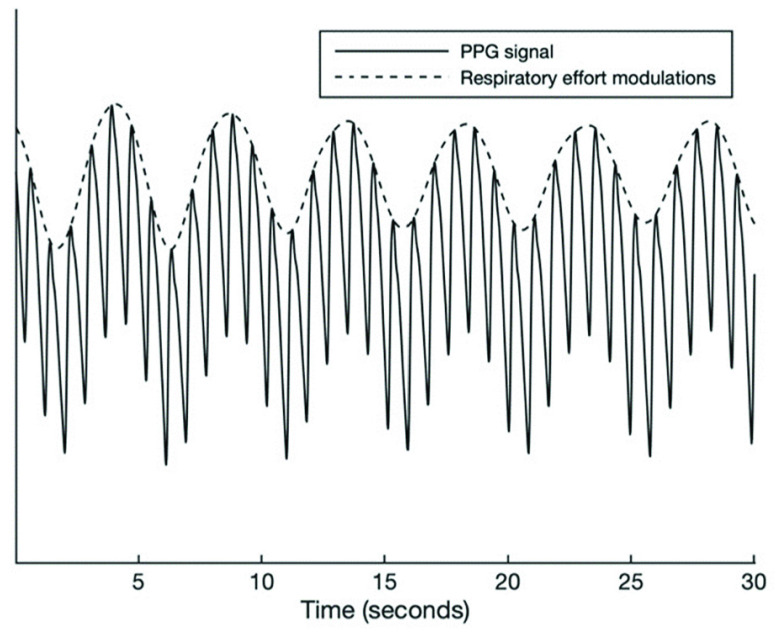
Example segment of PPG signal with the envelope reflecting respiratory effort modulations. PPG = photoplethysmography.

**FIGURE 4.**
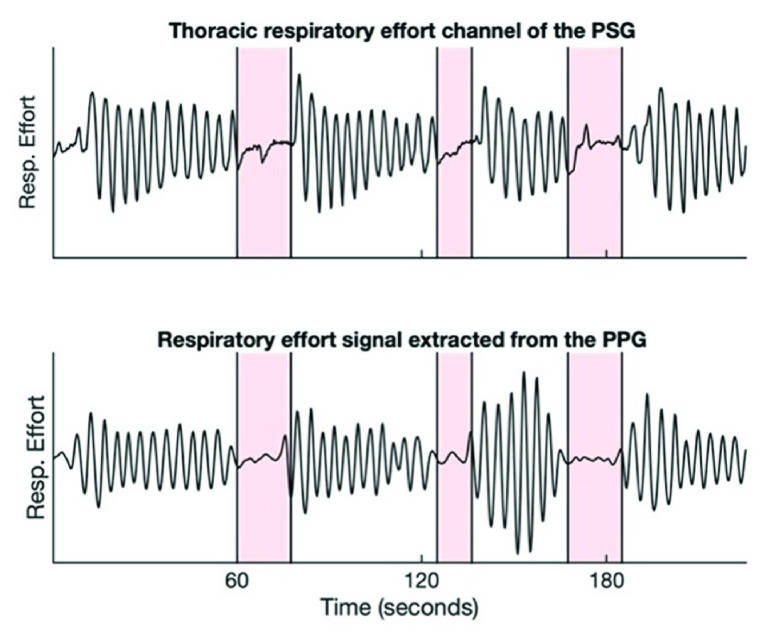
Comparison of respiratory effort signal extracted from the finger PPG with the respiratory effort channel of the PSG. Zones highlighted in pink indicate flat respiratory effort zones. PPG = photoplethysmography, PSG = polysomnography.

The PPG signal measured at the fingertip inherently contains respiratory information because the blood flow to body extremities gets affected by alterations in thoracic pressure throughout the respiratory cycle [19], [20]. Therefore, the PPG signal amplitude oscillates in synchrony with the respiratory cycle. This amplitude modulation can be isolated to retain a signal representing respiratory effort. Fig. 3 shows the PPG signal along with the respiratory modulations present in the signal. Our method utilizes the presence or absence of these breathing modulations in the finger PPG to infer the type of a respiratory event. The respiratory modulation was extracted from the raw PPG signal by means of the steps described below. The raw PPG signal was high-pass-filtered at 0.15 Hz to remove the slowly varying baseline modulations. The breathing frequency at rest is usually 0.2 Hz or higher (the normal breathing frequency range for an adult is 0.2-0.33 Hz) and, therefore, the breathing modulations remain substantially unaffected by such filtering. Subsequently, the peak envelope of the filtered PPG signal was calculated, which was further normalized by subtracting a 6-second moving average of this envelope and dividing it by a 30-second moving interquartile range operation. The 6-second window was chosen to remove signal baseline fluctuations while retaining breathing modulations, whereas the 30-second window was chosen to normalize any steady-state changes in the signal amplitude. The resulting signal, the PPG-derived respiratory effort (PPGDR) signal, was used for further analysis. Fig. 4 compares a segment of the PPGDR to the corresponding respiratory effort segment from the thoracic effort belt of the PSG.

#### 3) Feature Calculation

After PPGDR signal extraction, features were extracted from the PPGDR using a sliding-window operation. Two window sizes were used: 11 and 21 seconds. The window shifted over the PPGDR signal with a stride of 1 second and the feature values calculated for each window were ascribed to the one-second interval at the center of the window.

The features computed for each window were the range, the interquartile range, the variance, and the number of PPGDR local maxima (peaks). The range of the signal was additionally computed for window sizes of 5 and 7 seconds.

For local maxima-related features, local maxima detection was performed on the PPGDR (minimum peak-to-peak distance was set to 250 milliseconds), where the peak-to-peak interval represents the duration of a breathing cycle. A lack of local maxima suggests a lack of breathing effort.

In a next step, features were ascribed to each respiratory event detected by the PAT HSAT. Successful feature design must account for the variability in duration and location of the flat zones in the PPGDR during a respiratory event. As such, for each respiratory event detected by the PAT HSAT, the moving-window features for which the center-second overlapped with the respiratory event period were selected. From these selected features, the minimum value, the average of the lowest three values, and the $10^{\mathrm {th}}$ and $25^{\mathrm {th}}$ percentiles of the feature values were calculated.

For local maxima-related features, the aggregation operations comprised computing the minimum value and the numbers and proportions of windows with zero, one, and two peaks. The resulting aggregated feature sets for each PAT HSAT respiratory event were used to support subsequent steps.

#### 4) Labelling Respiratory Events

For model training purposes, each respiratory event inferred by the PAT HSAT was labeled using the Expert Analysis’ event type labels (central vs. obstructive). Since for model training we only wanted to retain respiratory events that were identified with a comparatively high level of certainty, respiratory events identified by the Expert Analysis that were not also identified by Local Analysis were removed. The respiratory events for which there was consensus by the Expert and Local Analysis on their presence (but not necessarily on their type) were used to label PAT HSAT events (Ground Truth Events). The PAT HSAT events that did not overlap with or were within ten seconds after any Ground Truth Event were removed from the training data. This removal of Expert Analysis or PAT HSAT events was only performed for the training sets and not for model validation. Each Expert Analysis’ annotation was used at most once for PAT HSAT event labeling.

**FIGURE 5.**
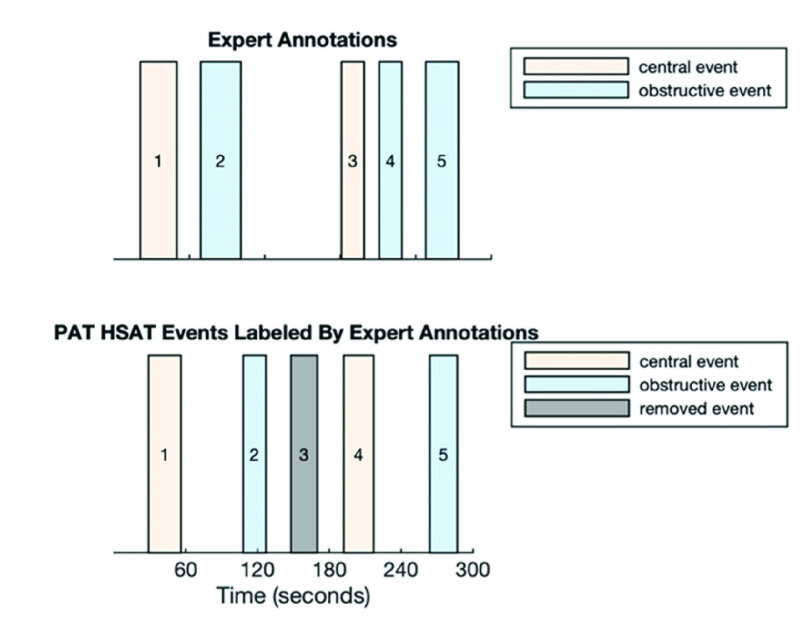
The procedure used to label PAT HSAT events. PAT HSAT event 1 is labeled as central because it has a uniquely overlapping central Expert annotation. PAT HSAT event 2 is labeled as obstructive because it has an Expert obstructive annotation preceding by not more than 10 seconds. PAT HSAT event 3 is removed because it cannot be labeled using the Expert annotations. This event will not be used for training. PAT HSAT event 4 has two overlapping annotations, but since at least one of them is central, it is labeled as central. PAT HSAT event 5 is labeled as obstructive because it has a single overlapping obstructive annotation. PAT HSAT = home sleep apnea testing based on peripheral arterial tonometry.

Fig. 5 illustrates the labeling procedure by means of examples. If a PAT HSAT event had a uniquely overlapping Expert Analysis annotation, the PAT HSAT event was labeled according to the corresponding Expert Analysis’ event type. If there was more than one annotated event overlapping with a PAT HSAT event, the PAT event was labeled as central if any of the overlapping Expert Analysis’ annotations were central events, otherwise the respiratory event was labeled as obstructive. Since the PAT HSAT respiratory event locations may be slightly shifted with regards to Expert Analysis’ annotations, any preceding Expert Analysis’ annotations of up to ten seconds were matched to a PAT HSAT event if there was no other annotation overlapping with the PAT HSAT event. Each Expert Analysis’ annotation was used at most once for PAT HSAT event labeling. Out of a total of 44,420 PAT HSAT events, 26,208 had a corresponding Expert Analysis annotation and were labeled according to the above-described procedure. Out of the 26,208 labeled PAT HSAT events, 2,881 were central (or mixed) and 23,327 were obstructive.

Since an airflow channel is necessary to mark the exact start and end of a respiratory event, a PAT HSAT has an imperfect delimitation of respiratory events. As such, it is not readily possible to infer via a PAT HSAT whether an episode without breathing effort followed by an episode with breathing effort corresponds to a central or a mixed event. For that reason, the Expert Analysis labels were binarized into central and obstructive, considering mixed events as central. The same approach is used for the Local Analysis labels. Therefore, the reported cAHI of our method is an estimate of the sum of central and mixed respiratory events per hour of sleep.

#### 5) Signal Rejection

**FIGURE 6.**
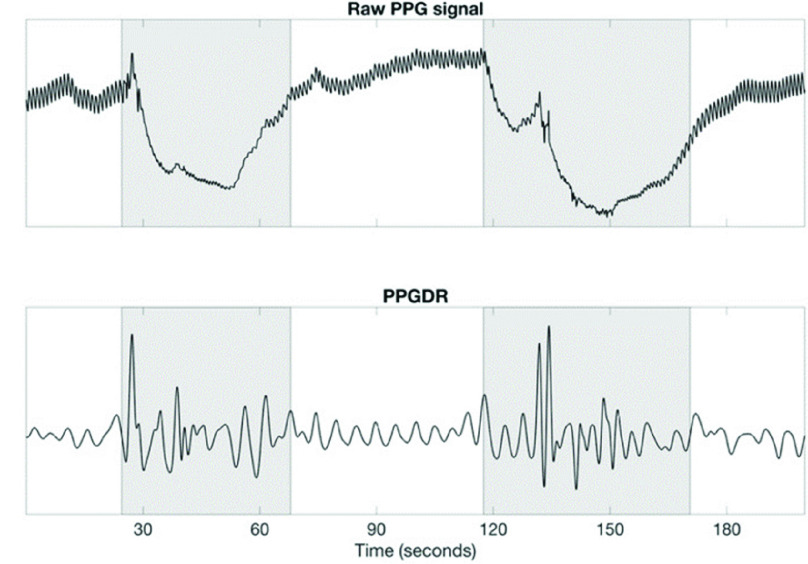
PPG signal and PPGDR of two vasoconstriction-affected zones (visualized by grey areas). The PPGDR extracted from these zones is of lower quality, apparent from the comparatively more erratic swings in the PPGDR signaland is therefore rejected. PPG = photoplethysmography, PPGDR = PPG-derived respiratory effort.

Because we use only PPG to extract the respiratory signal, the quality of our predictions is heavily dependent on the underlying PPG data quality. Strong vasoconstrictions reflected in sudden drops in PPG pulse amplitude frequently contain low signal-to-noise ratio episodes and do not get sufficiently canceled out by normalization operations. This significantly reduces PPGDR quality. We recognize vasoconstrictions by identifying troughs of significant prominence in a filtered version of the PPG signal. The filtering is performed to remove baseline shifts and shifts in perfusion that might otherwise impact trough detection. Fig. 6 shows an example of such a vasoconstriction-affected zone.

PPGDR extracted from such vasoconstriction-affected zones was rejected. That is, if an event had a vasoconstriction-affected zone, features from that zone were not used for the compilation of the aggregated feature set for this event.

#### 6) Event Rejection

Another issue with PPGDR signal quality arises when motion or other artifacts impact the PPG quality or when severe cardiac arrhythmia are present. When the peak-to-peak intervals of the PPG are irregular, either due to artifacts or severe cardiac arrhythmia, the breathing modulations present in the PPG get overpowered by such irregularities and PPGDR extracted from these regions would show spurious oscillations unrelated to the true respiratory modulations.

**FIGURE 7.**
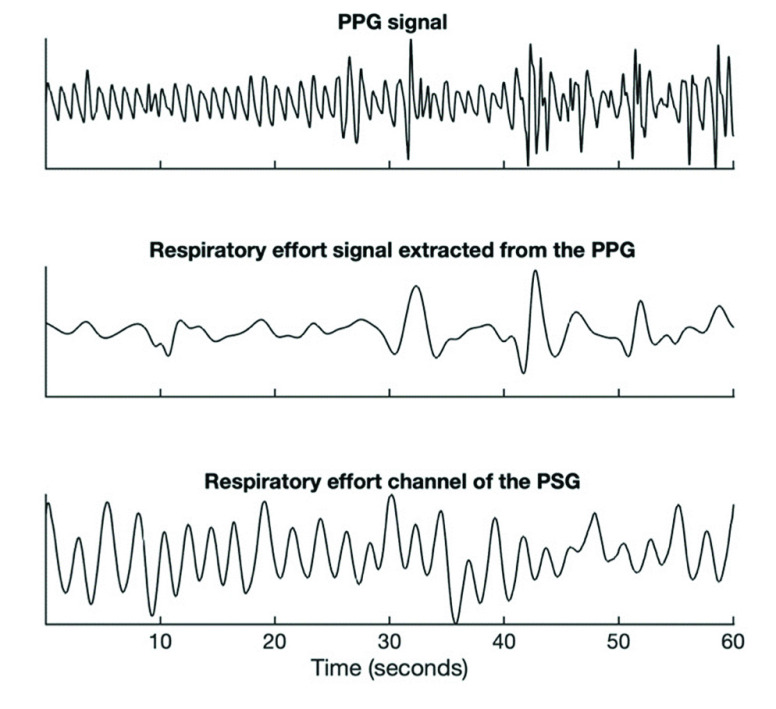
Example of an artifacted PPG signal (top), PPGDR extracted from the PPG signal (middle), and the corresponding respiratory effort channel of the PSG (bottom). The PPGDR extracted from this artifacted zone does not reflect the true respiratory modulations. PPG = photoplethysmography, PPGDR = PPG-derived respiratory effort, PSG = polysomnography.

To make sure that the PPGDR can be utilized for respiratory event type prediction, irregular PPG peak-to-peak interval zones were detected, and any respiratory events that had more than 20% of irregular PPG peak-to-peak intervals were rejected. Peak-to-peak intervals were labeled as irregular when the absolute value of the difference of two neighboring peak-to-peak intervals was higher than 20% of the average of those neighboring intervals. Fig. 7 shows a segment of a rejected PPG signal and the corresponding PPGDR compared against the respiratory effort channel of the PSG. The proportion of rejected respiratory events for each recording was called the Event Rejection Proportion (ERP).

#### 7) Patient Rejection

Predictions made for patients that have a substantial number of rejected events would be unreliable due to the small proportion of retained events used for cAHI estimation. Therefore, patients with an ERP higher than 0.3 were rejected and removed from further analysis.

#### 8) cAHI Prediction and Evaluation

Unlike for training, for evaluation purposes all 44,420 PAT HSAT events were retained regardless of the existence of a corresponding PSG annotation. To maximize utilization of the strongly imbalanced dataset caused by the low prevalence of CSA, training and evaluation were performed via a patient-wise leave-one-out cross-validation. For each patient, an ensemble of trees classifier (ensemble method: bagging, false positive cost: 2.5, number of trees: 30, max. number of splits: 150) was trained on the labeled data of the remaining patients. The model was insensitive to a wide range of tree and split numbers, hence no extensive hyperparameter optimization was performed. The false positive cost parameter of the classifier was used to tune the event-wise sensitivity and specificity of the predictions, used in this case to prevent over-detection of CSA. Using this classifier, predictions were made on the non-rejected PAT HSAT events of the considered patient. The predicted cAHI was calculated as the number of central events as predicted by the model, divided by the PAT HSAT Total Sleep Time (TST) estimate. Since predictions were made only with non-rejected events, the predicted cAHI was extrapolated by dividing it by one minus the ERP to account for the total number of respiratory events (rejected and non-rejected) of the patient.

Performance of the method was evaluated for cAHI cutoffs of 5, 10, and 15. The evaluation was performed on a patient-wise base by flagging a patients’ SA as central if the cAHI was above the given cutoff value.

### D. Statistical Analysis

#### 1) General

Statistical analysis was performed using MATLAB (version 2020a, MathWorks, USA). Patient demographic information was obtained during the clinical study and is reported in Results. For the cAHI predictions, the Pearson correlation with the ground truth was computed. For each of the cAHI cutoffs, cohort-wise sensitivity, specificity, positive and negative Likelihood Ratios (LR+, LR-), NPV, PPV, accuracy and Cohen’s Kappa values were computed, and a Receiver Operating Characteristic (ROC) and Precision Recall (PR) curve was generated. The ROC and PR curves were generated by obtaining classifier probability scores for each respiratory event and varying the probability cutoff threshold for binary classification. For all sensitivity, specificity, LRs, PPV, NPV, accuracy and Cohen’s Kappa endpoint parameters and 95% confidence intervals were computed. The classifier confusion matrix was constructed for four cAHI categories: cAHI < 5, $5\le $ cAHI < 10, $10\le $ cAHI < 15, and cAHI $\ge15$. Significance levels were determined for an alpha (p-value) of 0.05.

Where possible, the same analysis was performed for the comparison between the Local Analysis and the Expert Analysis. To estimate the importance of each feature, we summed changes in the risk due to splits on every feature and divided the sum by the number of branch nodes.

#### 2) Endpoint Analysis

Since there are no standardized performance targets established for cAHI prediction, the Local Analysis’s performance against the Expert Analysis serves as a performance benchmark. In line with Pillar et al., cAHI cutoffs of 10, and 15 were used for performance analysis. AHI cutoffs of 5 and 30 are also standard in literature, therefore performance analysis was also reported for the cAHI cutoff of 5.

As there were only 6 patients with a cAHI $\ge30$, no analysis was performed for this cutoff since the performance measures would be hard to generalize due to the small sample size.

The endpoints used for performance assessment were the sensitivity, specificity, LRs, NPV, PPV, accuracy, and Cohen’s Kappa of the PPG-based method at the examined cAHI cutoff values.

## IV. Results

### A. Patient Rejection

After removing patients with an ERP above the 0.3 cutoff from further analysis, 245 of the original 266 patients were retained. All statistical analyses and evaluations were performed on this final set of accepted patients.

### B. Demographic Information

As listed in Table 1, patients were predominantly male (60%), of middle age (mean 53.9 years, STD 13.7), and overweight (mean BMI 29.9 kg/m2 and STD 5.9). The mean AHI was 31.1 (STD 24.8).

**TABLE 1:** Demographic and Clinical Characteristics of Patients in Dataset

	Mean	STD	Min	Max
Age (years)	53.9	13.7	21.0	84.0
AHI (events/hr)	31.1	24.8	0.0	117.3
cAHI (event/hr)	3.2	7.5	0.0	45.5
BMI (kg $\text{m}^{\mathbf {-2}}$)	29.9	5.9	18.2	53.8

AHI = apnca-hypopnea index, cAHI = central apnea-hypopnnea index, BMI = body mass index, Max = maximum, Min = minimum, STD = standard deviation.

To investigate rejection bias related to skin pigmentation, which is a known source of PPG signal quality deterioration [27], the patient population is divided into 2 skin pigmentation categories. Category 1 consists of 186 patients with light skin pigmentation (Fitzpatrick Scale below III), while category 2 consists of 79 patients with darker skin pigmentation (Fitzpatrick Scale of III and above). For one patient the skin pigmentation information was not registered. The rejection ratios for categories 1 and 2 were 8.1% and 7.6% respectively. As such, no clear rejection bias related to skin tone was found. 24 patients had no clinical sleep apnea (AHI < 5), 56 patients had mild sleep apnea ( $5\le $ AHI < 15), 64 patients had moderate sleep apnea ( $15\le $ AHI < 30) and 101 patients had severe sleep apnea (AHI $\ge30$). 213 patients had cAHI < 5, 11 patients had $5\le $ cAHI < 10, 10 patients had 10 $\le $ cAHI < 15, 5 patients had 15 $\le $ cAHI < 30, and 6 patients had cAHI $\ge30$.

### C. Cohort-Wise Performance

**FIGURE 8.**
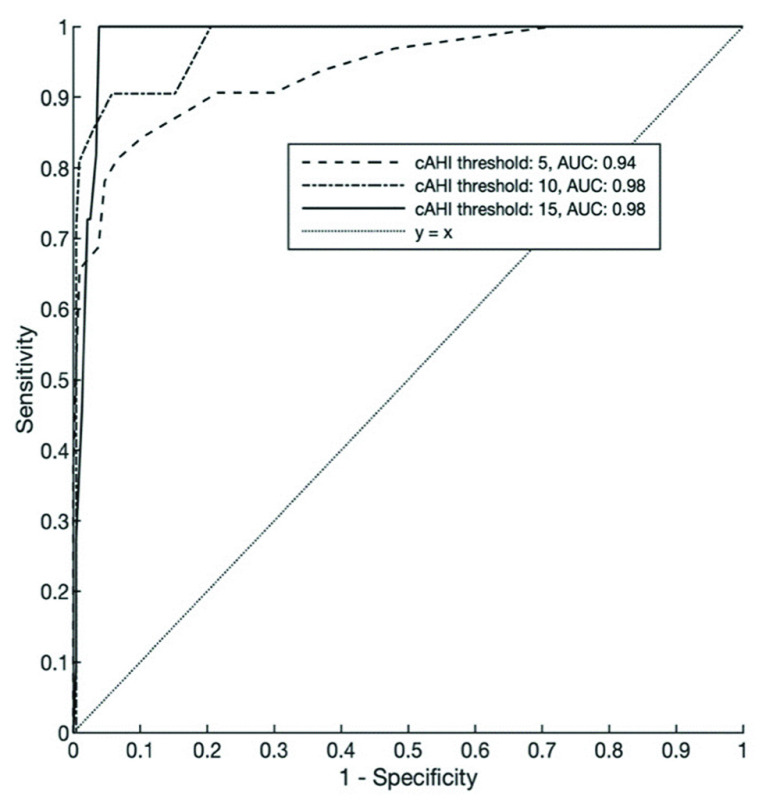
ROC curves for cAHI cutoffs of 5, 10, and 15 of the PPG-based method. cAHI = central apnea-hypopnea index, AUC = area under the curve, ROC =receiver operating characteristic.

**FIGURE 9.**
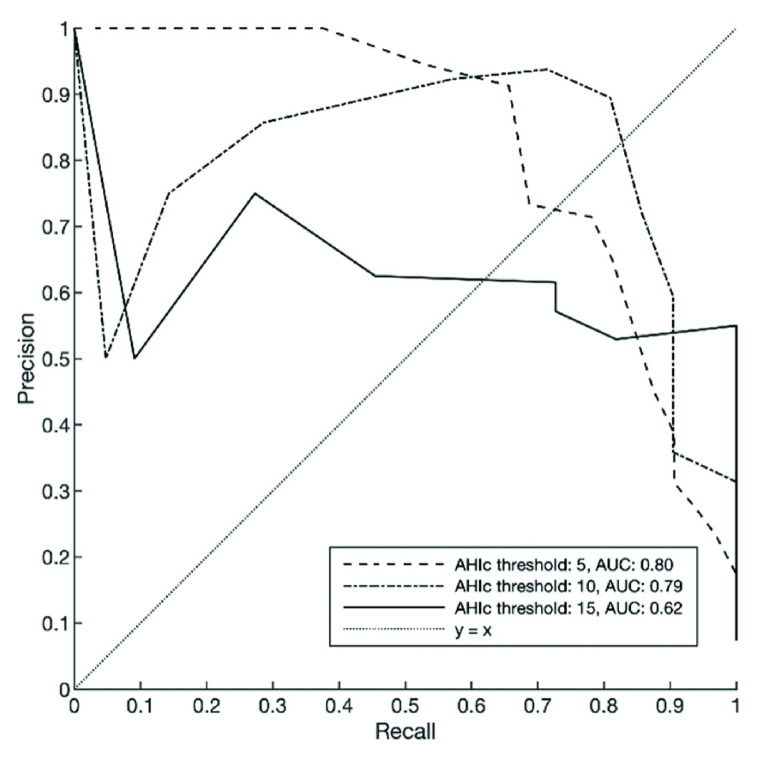
PR curves for cAHI cutoffs of 5, 10, and 15 of the PPG-based method. cAHI = central apnea-hypopnea index, AUC = area under the curve, PR = precision recall.

Table 2 shows the sensitivity, specificity, LRs, NPV, PPV, accuracy, and Cohen’s Kappa for three cAHI cutoffs for the PPG-based method against the Expert Analysis. The same is displayed for the Local Analysis against the Expert Analysis. For a cAHI cutoff of 10, the method achieved a sensitivity of 81%, a specificity of 99%, an LR+ of 90.7, an LR- of 0.19, a PPV of 90%, an NPV of 98%, an accuracy of 97.6%, and a Cohen’s Kappa of 0.84. Fig. 8 and Fig. 9 respectively show the ROC and the PR curves for each of the cAHI cutoffs. The largest ROC AUC of our method of 0.98 was found at a cAHI cutoff of 15. The largest PR AUC of 0.80 was found at a cAHI cutoff of 5.

**TABLE 2:** Performance Endpoints

cAHI cutoff		cAHI $\ge5$	cAHI $\ge10$	cAHI $\ge15$
Sensitivity (%)	PPG – EXPERT	78.1 [63.8, 92.4]	81.0 [64.2, 97.8]	72.7 [46.4, 99.0]
	LOCAL – EXPERT	71.9 [56.3, 87.5]	57.1 [35.9, 78.3]	72.7 [46.4, 99.0]
Specificity (%)	PPG – EXPERT	95.3 [92.5, 98.1]	99.1 [97.9, 100.0]	97.8 [95.9, 99.7]
	LOCAL – EXPERT	95.3 [92.5, 98.1]	99.1 [97.9, 100.0]	98.3 [96.6, 100]
LR+	PPG – EXPERT	16.6 [8.8, 31.3]	90.7 [22.5, 365.9]	34.0 [13.3, 87.1]
	LOCAL – EXPERT	15.3 [8.1, 29.1]	64.0 [15.3, 267.1]	42.5 [15.1, 120.0]
LR-	PPG – EXPERT	0.23 [0.12, 0.44]	0.19 [0.08, 0.46]	0.28 [0.11, 0.73]
	LOCAL – EXPERT	0.30 [0.17, 0.51]	0.43 [0.26, 0.71]	0.28 [0.11, 0.73]
PPV (%)	PPG – EXPERT	71.4 [56.4, 86.4]	89.5 [75.7, 100.0]	61.5 [35.0, 88.0]
	LOCAL – EXPERT	69.7 [54.0, 85.4]	85.7 [67.4, 100.0]	66.7 [40.0, 93.4]
NPV (%)	PPG – EXPERT	96.7 [94.3, 99.1]	98.2 [96.5, 99.9]	98.7 [97.2, 100.0]
	LOCAL – EXPERT	95.8 [93.1, 98.5]	96.1 [93.6, 98.6]	98.7 [97.2, 100.0]
Accuracy (%)	PPG – EXPERT	93.1 [89.9, 96.3]	97.6 [95.7, 99.5]	96.7 [94.5, 98.9]
	LOCAL – EXPERT	92.2 [88.8, 95.6]	95.5 [92.9, 98.1]	97.1 [95.0, 99.2]
Cohen’s Kappa	PPG – EXPERT	0.71 [0.57, 0.84]	0.84 [0.71, 0.97]	0.65 [0.41, 0.89]
	LOCAL – EXPERT	0.66 [0.52, 0.81]	0.66 [0.47, 0.86]	0.68 [0.45, 0.91]
ROC AUC	PPG – EXPERT	0.94	0.98	0.98
	LOCAL – EXPERT	NA	NA	NA
PR AUC	PPG – EXPERT	0.80	0.79	0.62
	LOCAL – EXPERT	NA	NA	NA

95% confidence interval estimates for the performance endpoints are shown in brackets. PPG – EXPERT refers to the PPG-based method’s predictions compared to Expert Analysis. LOCAL – EXPERT refers to the Local Analysis compared to the Expert Analysis. cAHI = central apnea-hypopnea index. AUC = area under curve, NPV = negative predictive value, PPV = positive predictive value, ROC = receiver operating characteristic, PPG = photoplethysmography.

**FIGURE 10.**
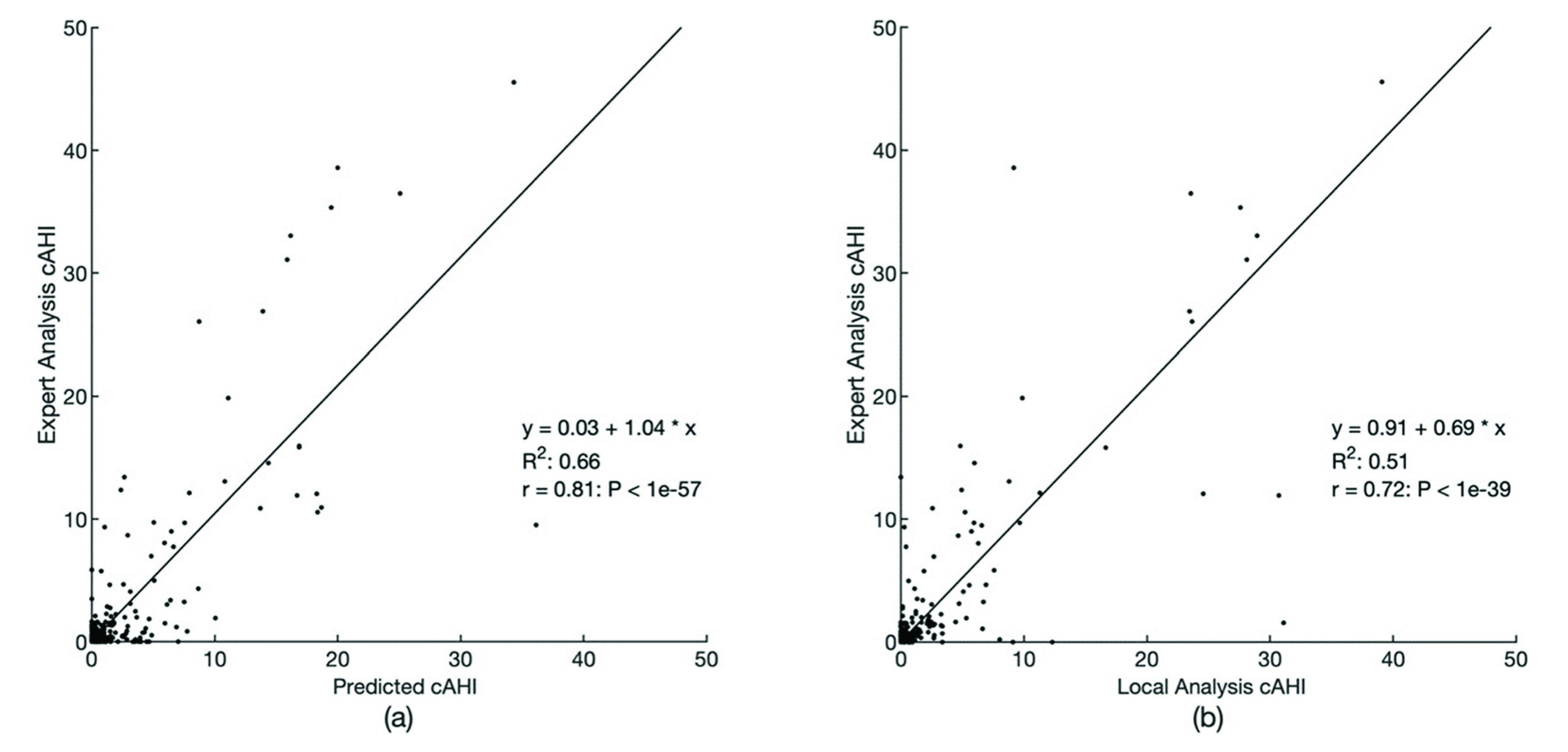
(Left) Scatterplot of Expert Analysis cAHI vs PPG-based method’s predicted cAHI. (Right) Scatterplot of Expert Analysis cAHI vs Local Analysis cAHI. cAHI = central apnea-hypopnea index, P: P-value, PPG = photoplethysmography, r: Pearson’s correlation coefficient, R^2^: R-squared, y = a $+\,\,\text{b}{}^{\ast} $ x: the equation of the least-squares fitted line.

Fig. 10 shows scatterplots of the cAHI predicted by the PPG-based method and Local Analysis’ cAHI against the Expert Analysis’ cAHI. The Pearson correlation was 0.81 for PPG-based predictions (p-value < 0.001) and 0.72 for the Local Analysis (p-value < 0.001). Table 3 shows the confusion matrix of PPG-based predictions for four cAHI intervals as well as the same analysis for the Local Analysis.

**TABLE 3:** Confusion Matrix

		Predicted Class							
		cAHI < 5		5 $\le $cAHI < 10		10 $\le $cAHI < 15		cAHI $\ge $15	
		PPG – EXPERT	LOCAL–EXPERT	PPG – EXPERT	LOCAL–EXPERT	PPG – EXPERT	LOCAL–EXPERT	PPG – EXPERT	LOCAL–EXPERT
True Class	cAHI < 5	203	203	9	8	1	1	0	1
	5 $\le $cAHI < 10	5	5	5	6	0	0	1	0
	10 $\le $cAHI < 15	2	3	1	3	3	1	4	3
	cAHI $\ge $15	0	1	1	2	2	0	8	8

PPG–EXPERT refers to the PPG-based method’s predictions compared to Expert Analysis. LOCAL–EXPERT refers to the Local Analysis compared to the Expert Analysis. cAHI = central apnea-hypopnea index, PPG = photoplethysmography

The 4-class accuracy for cAHI prediction derived from this matrix was 89.4% for PPG-based predictions and 89.0% for the Local Analysis. Cohen’s Kappa was 0.573 for PPG-based predictions and 0.544 for the Local Analysis.

### D. Feature Importance

Table 4 lists the 5 most important features which capture 53.1% of the total feature set importance. The best performing feature is the minimum range of the breathing signal calculated on a 7 second window sliding over the duration of the respiratory event with a shift of 1 s. The other features in the top 5 capture similar information. The local maxima features are the lowest ranked features.

**TABLE 4:** Ranked Feature Importance of Top 5 Features

	Feature importance (%)	Cumulative sum (%)
Range min (window $=7\text{s}$)	19.1	19.1
Variance min (window $=11\text{s}$)	12.4	31.5
IQR lowest 3 mean (window $=11\text{s}$)	7.7	39.2
Range min (window $=5\text{s}$)	7.0	46.2
Range percentile (window $=5\text{s}$)	6.9	53.1

## V. Discussion

### A. General

The method described in this paper allows for accurate cAHI prediction based on PPG data obtained by a finger probe and does so without the need for any additional sensor modalities such as a chest probe.

### B. Pat HSAT Performance

The performance of our method was found to be comparable to that of the Local Analysis of the concurrently acquired PSG.

Another comparison can be made between the proposed method and the PAT HSAT cAHI detection performance described by Pillar et al. [21]. (note that as stated earlier this method combines PPG and chest accelerometry modalities). The study population described by Pillar et al. [21] was preselected for CSA. As such, PPV and NPV endpoints could not be compared due the large prevalence discrepancies between their study and ours.

The method described by Pillar et al. [21] achieved a sensitivity of 71.4% and specificity of 98.6% for cAHI cutoff 10. For cAHI cutoff 15, it achieved a sensitivity of 66.7% and a specificity of 100%. In comparison, our method’s performance endpoints listed in Table 2 show an outperformance in the sum of sensitivity and specificity for both cAHI cutoffs.

### C. Selecting the cAHI Cutoff for CSA Screening; Clinical Implications and Tradeoffs

The choice of the most desirable cAHI cutoff value for CSA screening should take into consideration the tradeoff between NPV and PPV. Since patients flagged by the method will be referred to a full in-lab PSG, having a high PPV is important to avoid unnecessary follow-up examinations, whereas a high NPV is required to avoid false negative tests.

**FIGURE 11.**
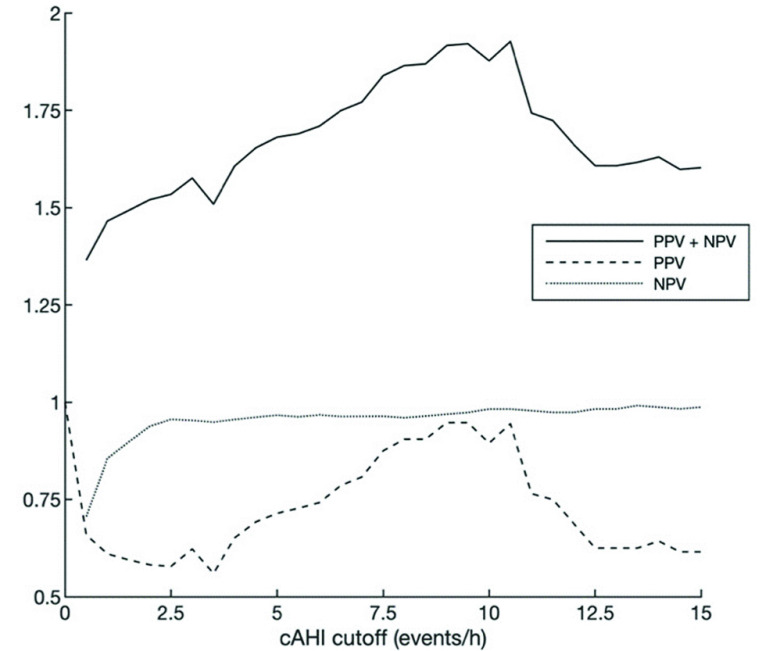
PPV, NPV, and the sum of their values for the PPG-based method for cAHI cutoffs in the range of 0 to 15. cAHI = central apnea-hypopnea index, PPV = Positive Predictive Value, NPV = Negative Predictive Value.

Fig. 11 shows the PPV, NPV, and their sum for varying cAHI cutoffs. For cAHI cutoffs between 2.5 and 15, the NPV remains relatively stable at around 95%, reaching a maximum of 99.1% at cAHI cutoff 13.5. The PPV increases from 57.8% at cAHI cutoff 2.5 to 94.7% at cAHI cutoff 9.5. At cutoff 10.5, it starts to decrease approximately monotonically. The cAHI cutoff corresponding to the highest sum of NPV and PPV of 1.93 was located at cAHI cutoff 10.5.

From Fig. 10 it can be observed that an underestimation bias is present for the PAT HSAT cAHI prediction for larger cAHI cutoffs (20 and above). However, the main goal of flagging potential central apnea patients is still achieved since each of the patients with cAHI $\ge30$ is categorized as cAHI $\ge15$ by the PAT HSAT.

Another consideration when selecting the cAHI besides maximizing NPV and PPV, is the selection of the desired sensitivity for detecting plausible CSA predominance. Predominant CSA occurs when 50% or more of the events are central in nature. As such, if we wish to detect patients with predominant CSA with an AHI as low as 5, we need to place the cAHI cutoff at 2.5. If we only wish to detect patients with predominant CSA with an AHI of 15 or higher, we need to place the cAHI cutoff at 7.5. The most performant cAHI cutoff of approximately 10 is optimized to flag predominant CSA in patients with an AHI of 20 or higher. Considering the above, the graph in Fig. 11 reveals the flexibility available to clinicians to select the cAHI cutoff based on this desired minimum AHI for which predominant CSA can be flagged. In the range of cAHI cutoffs between 2.5 and 10, a lower cAHI cutoff is generally associated with a lower PPV, and as such a higher rate of unnecessary follow-up examinations.

## VI. Limitations and Future Work

One limitation of this study was the low number of predominant CSA patients (only 4 patients in the study dataset) and CSA patients with cAHI $\ge30$ (only 6 patients in the study dataset). The limited sample made the study unreliable in analyzing the method’s performance of CSA predominance or cAHI $\ge30$ prediction.

Another limitation is that, although the study population matches the intended target population of the Study Device, some anthropometric and medical background data is missing. For instance, no fine-grained Fitzpatrick scales and no information on present comorbidities and medication use of subjects is available. This limits the generalizability of the research findings.

Future work should address these limitations by externally validating the method on a study population with a higher incidence of severe CSA and a more comprehensive medical background record. This will allow to analyze whether performance degradation may occur in certain subpopulations.

Aside from additional external validation, another possible improvement of the method would be utilizing multi-night PAT HSAT data for a more accurate CSA prediction.

Taking advantage of multi-night data might also decrease the number of patient recordings which were rejected due to large event rejection proportions.

## VII. Conclusion

This work presents a novel cAHI prediction method that uses only finger photoplethysmography data. The results suggest that this method could be incorporated into PAT HSAT for minimally invasive CSA screening. In this study population of patients with a suspicion of SA, the method performed similarly to the inter-rater variability of the cAHI estimation of PSG.
